# Psychometric Properties of the Zarit Burden Interview in Informal Caregivers of Persons With Intellectual Disabilities

**DOI:** 10.3389/fpsyg.2022.792805

**Published:** 2022-03-09

**Authors:** Alicia Boluarte-Carbajal, Rubí Paredes-Angeles, Arnold Alejandro Tafur-Mendoza

**Affiliations:** ^1^Facultad de Ciencias de la Salud, Universidad César Vallejo, Lima, Peru; ^2^Grupo de Estudios Avances en Medición Psicológica, Universidad Nacional Mayor de San Marcos, Lima, Peru; ^3^Instituto Peruano de Orientación Psicológica, Lima, Peru; ^4^Research Center (CIUP), Universidad del Pacífico, Lima, Peru

**Keywords:** Zarit Burden Interview, ZBI, caregivers, intellectual disabilities, Rasch analysis, psychometric properties

## Abstract

Intellectual disability leads to a loss of autonomy and a high level of dependence, requiring support from another person permanently. Therefore, it is necessary to incorporate the assessment of caregiver burden in healthcare actions, to avoid putting the health of caregivers and patients at risk. In this sense, the study aimed to analyze the internal structure of the Zarit Burden Interview (ZBI) in a sample of caregivers of people with intellectual disabilities, to provide convergent and discriminant evidence with a measure of the risk of maltreatment, and to estimate the reliability of the scores from the Classical Test Theory and the Rasch Measurement Theory. The study was instrumental. The sample consisted of 287 Peruvian informal primary caregivers of persons diagnosed with intellectual disabilities. To collect validity evidence, the internal structure (confirmatory factor analysis, CFA) and the relationship with other variables (convergent and discriminant evidence) were used, while reliability was estimated through the omega coefficient and Rasch analysis. The internal structure of the ZBI corroborated a unidimensional structure. In terms of convergent and discriminant evidence, the scale presents adequate evidence. Reliability levels were also good. Previously, the psychometric properties of the ZBI have not been studied in caregivers of people with intellectual disabilities, and it represents the first study of the scale in Peru. The results obtained will allow the use of this scale to design actions in the work with caregivers and studies to understand the psychology of the caregiver.

## Introduction

The concept of disability has evolved historically, before linked to a bio-medical model of disease and now based on a systemic model, and can be conceptualized as a health condition determined by the social context (World Health Organization [WHO], 2007). In this sense, the degree of disability depends on the interaction that the person establishes with others, being many times the attitudes of the environment an obstacle to personal, family, educational, social, or labor participation (Cuenot, 2018). It is estimated that 15.6% of people over 15 years of age live with a disability, of which 5.1% are under 14 years of age and 0.7% have a severe disability (World Health Organization [WHO] and World Bank, 2011). Disability in Peru represents 10.3% of the total population, of which, 48.3% present visual disability, 7.6% hearing, 3.1% to speak or communicate, 15.1% to move or walk, 4.2% to understand or learn, 3.3% to relate to others, and those with multi disability represent 18.4% (Instituto Nacional de Estadística e Informática [INEI], 2019). However, there is no exact statistics on disability due to intellectual deficit.

Intellectual disability is one of the disabilities that generates greater dependence on its environment and is commonly associated with other comorbidities, such as cerebral palsy, autism, seizures, and sensory problems (Uzun et al., 2020). According to Schalock et al. (2021), it is defined as a deficit in intellectual functioning, which is expressed through an intelligence quotient (IQ) below 70, in addition to limitations in social adaptation skills, such as self-direction, social skills, movement, and personal care among others, presenting before the age of 18 years (Tassé et al., 2016). This type of disability leads to a loss of autonomy and a high level of dependence, requiring support from another person permanently, commonly referred to as a caregiver (Seguí et al., 2008).

According to the literature, there are two types of caregivers: formal and informal. The formal caregiver is the person who receives payment for the service. While the informal caregiver is commonly a family member, friend, or neighbor. Although this type of caregiver is neither paid nor trained to perform the work, they have a high degree of commitment characterized by their affective bond (Ruiz and Nava, 2012; Montero et al., 2014).

In the case of people with intellectual disabilities, the type of care in healthcare is usually informal, with the mother being the main caregiver (Boluarte, 2019). Although currently there is greater economic and social participation of women. However, still retains the traditional role in child-rearing (Duarte and García-Horta, 2016; Tartaglini et al., 2020). In turn, other family members also assume the role of caregiver, such as grandparents, siblings, and in some cases the father, who, in light of the existing literature, assumes little responsibility for the child-rearing (Espín, 2008; Lima-Rodríguez et al., 2018; Rodríguez et al., 2019). In this context, the presence of dysfunctionality in family relationships, inadequate coping styles, job abandonment, and low educational level of the mother not only affects the quality of family life (Espín, 2008; Cohen et al., 2014) but also the deterioration of physical, mental, and social health with serious psychopathological implications (Seguí et al., 2008).

In this way, caregiver burden has been studied, defined as the attitudes and emotional-affective reactions from the experiences of the role with repercussions in the personal, family, and social spheres (Zarit et al., 1980). Therefore, it is necessary to incorporate the assessment of caregiver burden in healthcare actions, to avoid putting the health of caregivers and patients at risk (Zarit et al., 1986; Schreiner et al., 2006).

There are different scales to measure caregiver burden (Crespo and Rivas, 2015), such as the Caregiver Burden Inventory (CBI; Novak and Guest, 1989), widely used in a different diagnostic groups, in children, adolescents, and adults with spinal cord injury (Farmer et al., 2018; Conti et al., 2019; Ortiz-Rubio et al., 2021). The CBI has been recently adapted to Spanish (Vázquez et al., 2019) obtaining a reduced version of 15 items. Likewise, the Screen for Caregiver Burden (SCB; Vitaliano et al., 1991), is used for the objective and subjective measurements of caregiver burden in the elderly.

One of the most widely used instruments is the Zarit Burden Interview (ZBI; Zarit et al., 1980). This measure, unidimensional and with 29 items in its original version, assesses the caregiver’s health, psychological wellbeing, finances, social life, and the relationship between the caregiver and the person with a disability. However, its factor structure and extent have had modifications over time (Knight et al., 2000; Yu et al., 2018).

The 22-item version (Zarit et al., 1985) has been studied in different contexts and translated into different languages, such as the United Kingdom (Siegert et al., 2010), Turkey (Özlü et al., 2009), Singapore (Seng et al., 2010), Portugal (da Cruz, 2010), Brazil (Taub et al., 2004), and among others. The adaptation to Spanish was carried out by Martín-Carrasco et al. (1996) and later re-evaluated by Martín-Carrasco et al. (2010), who showed that this measure was made up of three factors (i.e., burden, competence, and dependence) showing correlation with the caregiver’s mental health status and the presence of behavioral disorders in the patient.

In its factorial structure, it has been found the presence of three factors with different denomination: impact of care, interpersonal burden, and self-efficacy expectations (Montorio et al., 1998); shame, anger, and self-criticism (Knight et al., 2000); tensions referring to the role, intrapsychic tensions, competencies, and expectations (Bianchi et al., 2016); subjective impact, competence, and dependence (Martín-Carrasco et al., 2010); role-related strain, self-criticism, and negative emotion (Tang et al., 2016). Other studies report the presence of five factors: sacrifice, loss of control, shame/anger, self-criticism, and dependence (Lu et al., 2009), caregiver feeling of over-sacrifice, dependency, negative emotion, caregiver feeling of inadequacy, and uncertainty about the patient’s future (Ko et al., 2008). Other studies report four-factor structures: personal effort, privacy conflict, uncertain attitude, and guilt (Yoon and Robinson, 2005); consequences of caregiving on the caregiver, patient dependency, caregiving fatigue, uncertainty, guilt, and fear (Al-Rawashdeh et al., 2016); interpersonal burden, the impact of caregiving, competencies, and expectations about caregiving (Barreto-Osorio et al., 2015). The different theoretical interpretation in the measurement of caregiver burden with the ZBI is evident, demonstrating that there is no clear consensus on the dimensionality of the instrument (Monreal and Prieto, 2017).

Short versions are proposed to achieve a parsimonious structural model, which allows the instrument to be used in interventions that require optimizing the application time. In this sense, Hébert et al. (2000) with a 12-item version proposes a two-factor model: personal strain and role strain. Likewise, Bédard et al. (2001) proposed a short version of 12 and a 4-item version for screening studies, reporting its usefulness in caregivers of patients with different diagnoses (caregivers of older adults and children with chronic disabilities). The 12-item unidimensional version has shown adequate psychometric properties in several studies (O’Rourke and Tuokko, 2003; Ballesteros et al., 2012; Lin et al., 2017; Rueda et al., 2017; Pinyopornpanish et al., 2020; Tartaglini et al., 2020).

The literature shows that there is no clear consensus on the dimensionality of the instrument (Monreal and Prieto, 2017). While some studies state the presence of three dimensions, others report the presence of one or more additional factors. Li et al. (2018) named the fourth-factor as caregiving performance corresponding to items 20 and 21. However, Barreto-Osorio et al. (2015) indicate that these items would be related to indecisiveness about caregiving (7, 20, and 21). The conceptual controversies of the construct could be explained by a solid theoretical basis. The updated theory of attachment and emotional self-regulation allows understanding the conflicts in close relationships (Jarrett, 1985; Mikulincer and Shaver, 2005), caring for people dependent on the family context, generates positive and negative affective relationships, which alter the quality of the relationship with repercussions on the quality of family life.

Reliability has been commonly measured by internal consistency, in the case of global, measures the coefficients fluctuated between 0.70 and 0.93 (Lu et al., 2009; Özlü et al., 2009; Bianchi et al., 2016). While in multidimensional analysis, coefficients below 0.70 are reported (Hébert et al., 2000). Other methods, such as test-retest and inter-observer reliability, have also been used, using the interclass correlation coefficient (ICC) technique, obtaining good results, e.g., ICC = 0.88 (Taub et al., 2004) and ICC = 0.78 (Rajabi-Mashhadi et al., 2015) in samples of caregivers of chronically disabled patients.

On the other hand, studies show the relationship of caregiver burden with other variables, such as the risk of caregiver abuse (Özcan et al., 2017; Orfila et al., 2018; Saravia et al., 2019). A positive correlation has also been observed (*r* = 0.844; *p* < 0.001) with abuse of caregivers of people with behavioral disorders affected by dementia to a moderate degree (Gimeno et al., 2021), indicating that the higher the level of burden, the risk of maltreatment increases by the caregiver. Likewise, in people with dementia in Spain, a moderate relationship (*r* = 0.486; *p* < 0.001) was found between the risk of caregiver abuse, measured through the Caregiver Abuse Screen (CASE), and caregiver burden, assessed by the ZBI (Rivera-Navarro et al., 2018).

Most of the ZBI psychometric studies have been tested in samples of caregivers of adult patients with mental and degenerative diseases (e.g., dementia, Alzheimer’s, and acquired brain injury). To date, there are no psychometric studies that have assessed the ZBI in caregivers of people with intellectual disabilities. However, there are studies on the burden of caregivers in this population. Kim et al. (2021) found a partial mediation of family functioning on the relationship between care burden and quality of life for caregivers of children with intellectual disabilities in Mongolia. For the measurement of care burden, they used the CBI, reporting good levels of reliability (α > 0.70). In addition, Barros et al. (2019) found a negative relationship between quality of life domains (physical, psychological, social relationships, environment, and global) and the burden of caregivers of children and young adults with intellectual disabilities in Brazil. The assessment of burden of caregivers was conducted through the ZBI. However, due to the nature of the study, they only reported the overall estimate of reliability, which was good (α = 0.90).

The psychometric properties of the ZBI have not been examined in the Peruvian context, being relevant to know its psychometric properties to recommend its appropriate use in clinical-therapeutic assessment and intervention. Therefore, the present study is aimed to analyze the internal structure of the Zarit Burden Inventory in a sample of Peruvian primary caregivers of people with intellectual disabilities and to test the convergent and discriminant validity with a measure of the risk of maltreatment. In addition, we seek to estimate reliability using Classical Item Theory and Rasch Measurement Theory.

## Materials and Methods

### Study Design

The study was instrumental in that it analyzed the psychometric properties of a scale that measures caregiver burden (Ato et al., 2013). The development of the study followed the guidelines proposed in the standards for educational and psychological tests (American Educational Research Association [AERA] et al., 2014). Complementarily, recommendations based on good practices for the development and review of scales in social, health, and behavioral sciences were considered (Boateng et al., 2018).

### Participants

The selection of the participants was carried out through intentional non-probability sampling (Kerlinger and Lee, 2000). To determine the sample size, the recommendations for conducting a factor analysis were followed. In this sense, a ratio of three indicators per factor, a one-factor solution, low levels of communality, and an excellent-level criterion agreement (0.98), was considered *a priori*, obtaining a recommended sample size of 150 participants (Mundfrom et al., 2005). From this result, it was sought to obtain a sample size greater than the minimum recommended.

The initial sample size was 303. However, after the elimination of 16 outliers, the final study sample consisted of 287 informal primary caregivers of persons diagnosed with intellectual disabilities. The age of the caregivers ranged from 19 to 76 years (median = 40, median absolute deviation = 10.38). Regarding the characteristics of the caregivers, the majority were women (84.67%), the predominant marital status was married (40.21%), the highest proportion had a completed high school education (52.26%), had no illnesses (72.13%), were 24-h caregivers (45.64%), and most were fathers or mothers of the patients (86.76%). The persons with intellectual disabilities were aged between 1 and 64 years (median = 9, median absolute deviation = 4.45), and the highest percentage was moved without assistance (89.90%). A detailed description of the main demographic characteristics of the study participants is presented in Table 1.

**TABLE 1:** Sociodemographic characteristics of the participants (*n* = 287).

Variable	Category	Frequency	Percentage
Sex of caregiver	Male	44	15.33
	Female	243	84.67
Marital status	Single	52	18.18
	Married	115	40.21
	Cohabitant	92	32.17
	Separated	19	6.64
	Divorced	1	0.35
	Widower	7	2.45
Education level	Primary	25	8.71
	Secondary	150	52.26
	Technical superior	69	24.04
	Superior university	42	14.63
	No education	1	0.35
Caregiver with illness	Yes	80	27.87
	No	207	72.13
Relationship to patient	Parent	249	86.76
	Brother/sister	9	3.14
	Grandmother	14	4.88
	Uncle/aunt	7	2.44
	Another	8	2.79
Care hours	Between 1 and 5 h	21	7.32
	Between 6 and 10 h	43	14.98
	Between 11 and 15 h	28	9.76
	Between 16 and 20 h	29	10.10
	Between 21 and 24 h	35	12.20
	24 h a day	131	45.64
Main reason for caring	Own initiative	216	75.26
	Family decision	56	19.51
	Only one who could	15	5.23
Time as caregiver	Less than 1 year	17	5.92
	Between 1 and 3 years	32	11.15
	Between 3 and 6 years	62	21.60
	Between 6 and 9 years	62	21.60
	More than 10 years	114	39.72
Has another job	Yes	130	45.30
	No	157	54.70
Patient displacement	Moves with assistance	29	10.10
	Can move without assistance	258	89.90

### Measures

#### Zarit Burden Interview

This instrument was designed by Zarit et al. (1980) to measure the perception of primary caregiver strain. In this study, the version adapted to Spanish by Rueda et al. (2017) was used in a group of Colombian family caregivers. The ZBI is composed of 22 items that are answered on a five-point Likert-type scale (Never = 0; Rarely = 1; Sometimes = 2; Quite often = 3; and Almost always = 4). The ZBI items assess the perceived impact of caregiving on the caregiver’s physical health, emotional health, social activities, and financial situation. Overall ZBI scores range from 0 to 88 points, where a high score implies a greater perceived caregiver burden.

#### Caregiver Abuse Screen

This brief measure was designed by Reis and Nahmiash (1995) and is self-administered by caregivers. The CASE aims to detect the risk of physical, psychosocial abuse, and neglect by primary caregivers toward older adults (Reis and Nahmiash, 1995). For this study, the Spanish version of the CASE developed by Pérez-Rojo et al. (2015) was used. The CASE is made up of eight items grouped into two factors, Abuse (six items) and Neglect/Dependency (two items), which were found in the original study (Reis and Nahmiash, 1995) and the Spanish version (Pérez-Rojo et al., 2015). However, the Brazilian (Reichenheim et al., 2009) and Pakistani (Khan et al., 2020) versions suggest the presence of a unidimensional structure. The items are answered on a dichotomous response scale (No = 0; Yes = 1), so, their total scores vary between 0 and 8, where a higher score indicates a high risk of maltreatment.

In this study, for the collection of validity evidence based on internal structure, a two-factor related model was tested through confirmatory factor analysis (CFA), obtaining adequate fit indices [χ^2^ = 41.284, df = 19, χ^2^/df = 2.173, Comparative Fit Index (CFI) = 0.973, Tucker-Lewis Index (TLI) = 0.961, Root Mean Square Error of Approximation (RMSEA) = 0.064 [90% confidence interval (CI): 0.037, 0.091], Standardized Root Mean Square Residual (SRMR) = 0.070, and Weighted Root Mean Square Residual (WRMR) = 0.885]. However, scores on the Neglect/Dependency factor presented a low level of reliability (ω = 0.412), unlike the abuse factor (ω = 0.768), which obtained an acceptable level. Because of these results and the high correlation between the factors (*r* = 0.940), a unidimensional model was tested. The CFA indicated good fit indices for the unifactorial structure (χ^2^ = 41.381, df = 20, χ^2^/df = 2.069, CFI = 0.974, TLI = 0.964, RMSEA = 0.061 [90% CI: 0.034, 0.088], SRMR = 0.070, and WRMR = 0.889) and the factor loadings were found to be between 0.527 and 0.876. Likewise, the average variance explained (AVE) was 0.491, showing an acceptable level of convergent evidence. Regarding reliability, the CASE showed acceptable internal consistency (ω = 0.793 [95% CI: 0.733, 0.824]).

### Procedure

People with intellectual disabilities from special basic education schools and private psychological centers that serve this population were identified, and their main caregivers were identified. The identified caregivers signed an informed consent form, where the objective of the research was explained to them and the guarantee of confidentiality and anonymity of their participation. Subsequently, the caregivers proceeded to fill out the data collection form in person. This form consisted of a sociodemographic survey and the two measurement instruments (ZBI and CASE).

Once the database with 303 responses was obtained, univariate outliers were analyzed through the median absolute deviation (Leys et al., 2019) and multivariate outliers through the Mahalanobis-MCD distance (Leys et al., 2018). Four cases of univariate outliers and 12 cases of multivariate outliers were found, being removed from the database, leaving the final database with 287 participants. This process was carried out because many statistical procedures are affected by the presence of outliers. In some situations, in the presence of outliers, the statistical power of some methods presents less power and therefore, unreliable results (Aguinis et al., 2013). Likewise, outlier detection is a recommended good practice in data management. Finally, a sensitivity analysis was performed with and without outliers, observing a small impact of outliers, mainly in descriptive statistics, such as the mean (Thabane et al., 2013).

### Ethical Considerations

The present study was carried out with the commitment to ethical standards and values, where the researchers assume total responsibility and veracity demonstrating each result obtained, likewise, the reliability of the data has been acquired respecting the anonymity of the participants, in such a way that the personal information concerning those evaluated in the study is not known. The study was authorized by the ethics committee of the Universidad César Vallejo. Participants completed informed consent form to respond to the measurement instruments. All procedures performed in studies involving human participants were following the 1964 Helsinki Declaration and its later amendments or comparable ethical standards.

### Data Analysis

Statistical analyses were carried out in five stages. In the first stage, the descriptive measures of the items were obtained through the mean, standard deviation, skewness, and kurtosis. These last two coefficients indicated the level of departure from a normal distribution, considering adequate values between −2 and 2 (Tabachnick and Fidell, 2019). Likewise, the floor effect and ceiling effect of the items were analyzed, considering the percentage of people who answered the lowest and highest answer alternative, respectively. In this sense, those items with percentages equal to or less than 15% were evaluated as free of these effects (McHorney and Tarlov, 1995). Additionally, the discrimination of the items was estimated through the corrected item-rest polyserial correlation, taking as acceptable indices greater than 0.20 (Schmeiser and Welch, 2006).

In the second stage, validity evidence was collected based on the internal structure of the test using CFA. The estimation method was the Weighted Least Squares Means and Variance adjusted (WLSMV) with robust SEs and Scaling-Shifted scaled statistic test (SS), applied to the matrix of polychoric correlations of the items. To fix the metrics of the dimensions, one of their indicators, called the reference indicator, was used. That is, the factor loadings of the first indicator with its dimension were fixed. These fixed loadings were equal to 1. Regarding the goodness-of-fit indices to assess the estimated models, the ratio between Chi-square and degrees of freedom (SSχ^2^/df) was used, taking as appropriate values below 5 (Schumacker and Lomax, 2016); the CFI and TLI with adequate values higher than 0.90 (Keith, 2019); the RMSEA and SRMR considering values less than 0.08 as adequate (Schumacker and Lomax, 2016); and the WRMR with values lower than 1 being appropriate (DiStefano et al., 2018). Likewise, factor loadings above 0.40 were considered acceptable (Brown, 2015).

Later, in the third phase, validity evidence was collected based on the relationship with other variables. For this purpose, convergent and discriminant evidence was collected. The convergent evidence was evaluated from the average variance extracted (AVE), taking as minimum acceptable values those proposed by Moral (2019), which considers the factor loadings, the reliability coefficient, and the number of factor items evaluated. A structural equation model (SEM) was tested to estimate the relationship between ZBI and CASE, using the same criteria as in the CFA to assess model fit. In addition, the relationship between the variables was assessed as small, medium, and large considering correlation coefficients above 0.10, 0.30, and 0.50, respectively (Cohen, 1988). On the other hand, the discriminant evidence was collected through two procedures, the heterotrait-monotrait ratio (HTMT), taking as adequate values lower than 0.85 (Henseler et al., 2015), and the Fornell and Larcker criterion, which consists of comparing the square root of the AVE and the correlations with the other variables, where the former must be greater than the latter to conclude that there is discriminant evidence (Fornell and Larcker, 1981).

In the fourth phase of analysis, the reliability of the test scores was evaluated using the internal consistency method. For this objective, the ordinal omega coefficient was used, estimated from the factorial solution obtained from the CFA (Viladrich et al., 2017; Flora, 2020). This coefficient varies between 0 and 1, being valued as adequate from 0.70 (Nunnally and Bernstein, 1994). CIs were estimated at a 95% confidence level using the bias-corrected and accelerated bootstrap method with 10,000 replications. Additionally, to have a better understanding of the score reliability, inter-item polychoric correlations were estimated (Ventura-León and Peña-Calero, 2021).

Finally, an analysis of the ZBI was carried out from the perspective of Rasch Measurement Theory, using Andrich’s Rasch model or Rating Scale. Within this analysis, the functionality of the response categories was analyzed based on the use of statistical criteria for scale optimization. In addition, the fit of the model to the data was evaluated based on three fit indices (RMSEA, TLI, and CFI), taking as acceptable values those presented in the CFA. On the other hand, the reliability of the persons and items was estimated, considering values above 0.70 adequate (Nunnally and Bernstein, 1994). Finally, the fit of the items to the model was verified using the Infit and Outfit statistics, being considered good between 0.7 and 1.3 (Wright and Linacre, 1994; Bond and Fox, 2013).

The data analysis was carried out through R version 4.1.1 (R Core Team, 2021) in the RStudio graphical user interface (RStudio Team, 2021). The tidyverse package version 1.3.1 (Wickham et al., 2019) was used for data manipulation; the Routliers package version 0.0.0.3 (Klein and Delacre, 2021) for the identification of outliers; the psych package version 2.1.9 (Revelle, 2021) for descriptive analysis; the lavaan package version 0.6-9 (Rosseel, 2012) for the CFA; the semTools package version 0.5-5 (Jorgensen et al., 2021) to estimate the reliability, AVE, and HTMT; the CTT package version 2.3.3 (Willse, 2018) to estimate the polyserial item-rest correlation; the MBESS package version 4.8.0 (Kelley, 2020) for the estimation of CIs for reliability coefficients; and the mirt package version 1.34 (Chalmers, 2012) for Rasch analysis.

## Results

### Item Analysis

The descriptive statistics of the items (Table 2) indicated that item 20 presented the highest mean score (*M* = 2.78), while item 5 reported the lowest mean (*M* = 0.39). Regarding the variability of the responses, item 14 showed the highest (SD = 1.50) and item 5 the lowest (SD = 0.80). Regarding the levels of skewness and kurtosis, items 4 and 5 presented values higher than 2, indicating a slight distortion of the data concerning a normal distribution. On the other hand, most of the items presented floor and ceiling effects, where more than 15% of the responses were concentrated in response options 0 or 4. Regarding the discriminative ability of the items, all showed a polyserial item-rest correlation coefficient above 0.20, except item 20, which had a value slightly below the indicated criterion.

**TABLE 2:** Descriptive statistics, the proportion of responses, and discrimination of the items.

						Responses (%)				
Item	*M*	SD	Sk	Ku	Item-rest correlation	0	1	2	3	4
1	2.12	1.25	0.08	−0.91	0.43	10.80	19.86	36.59	12.20	20.56
2	1.56	1.36	0.39	−1.02	0.51	31.01	18.82	26.13	11.50	12.54
3	1.28	1.23	0.60	−0.58	0.69	36.93	19.86	28.57	7.67	6.97
4	0.48	0.94	2.08	3.81	0.53	73.52	12.20	9.41	2.44	2.44
5	0.39	0.80	2.24	4.88	0.51	76.31	12.54	8.36	1.74	1.05
6	0.55	0.97	1.65	1.76	0.54	70.38	11.15	12.54	4.53	1.39
7	2.20	1.43	−0.08	−1.29	0.44	15.68	17.42	27.18	10.45	29.27
8	2.40	1.35	−0.32	−1.06	0.36	11.85	13.59	26.48	18.47	29.62
9	0.66	1.05	1.53	1.52	0.62	64.81	13.94	14.63	3.48	3.14
10	0.64	1.02	1.47	1.24	0.59	66.20	12.20	15.33	4.18	2.09
11	0.72	1.03	1.38	1.25	0.57	58.54	19.86	15.33	3.48	2.79
12	0.71	1.10	1.45	1.12	0.63	63.76	13.94	13.59	5.23	3.48
13	0.55	0.93	1.63	1.93	0.55	68.29	13.94	13.24	3.14	1.39
14	1.97	1.50	0.06	−1.41	0.40	23.69	17.77	20.56	13.59	24.39
15	1.82	1.40	0.22	−1.18	0.56	23.00	20.91	25.44	12.20	18.47
16	0.91	1.11	1.06	0.28	0.29	49.48	23.00	18.12	5.92	3.48
17	0.67	1.02	1.42	1.07	0.62	63.07	16.38	13.24	5.57	1.74
18	0.62	0.99	1.50	1.45	0.25	66.20	12.54	16.38	2.79	2.09
19	1.01	1.17	0.89	−0.15	0.39	47.74	18.47	23.34	5.92	4.53
20	2.78	1.34	−0.84	−0.54	0.17	10.10	9.76	13.24	26.13	40.77
21	2.66	1.34	−0.65	−0.75	0.27	10.45	9.76	20.21	23.00	36.59
22	1.17	1.25	0.76	−0.40	0.63	42.86	17.42	26.83	5.23	7.67

*M = mean; SD = standard deviation; Sk = skewness; Ku = kurtosis.*

### Validity Evidence Based on the Internal Structure

Nineteen different models were tested that differed from each other in terms of the number of factors, number of items, and the ordering of items in the factor structures. Five models did not converge because their covariance matrix of latent variables is not positive definite: Özlü et al. (2009); Ko et al. (2008), Flynn and Knight (2011); Chattat et al. (2011), and James et al. (2021). Additionally, three models were not interpreted as some estimated observed variable variances are negative: Montero et al. (2014); Lu et al. (2009), Yoon and Robinson (2005). Thus, 11 models were estimated and interpreted (Table 3).

**TABLE 3:** Results of the confirmatory factor analysis.

Model	Factors	SSχ^2^	df	SSχ^2^/df	RMSEA [90% CI]	CFI	TLI	SRMR	WRMR
1. Ballesteros et al., 2012	1	156.361	54	2.896	0.081 [0.067, 0.096]	0.935	0.921	0.076	1.007
2. Bédard et al., 2001	2	320.371	53	6.045	0.133 [0.119, 0.147]	0.843	0.805	0.126	1.612
3. Tartaglini et al., 2020	1	356.772	119	2.998	0.084 [0.074, 0.094]	0.913	0.901	0.088	1.219
4. Martín-Carrasco et al., 2010	3	837.240	206	4.064	0.104 [0.096, 0.111]	0.806	0.782	0.118	1.683
5. Hébert et al., 2000	2	133.508	53	2.519	0.073 [0.058, 0.088]	0.962	0.953	0.064	0.893
6. Bianchi et al., 2016	3	869.989	206	4.223	0.106 [0.099, 0.113]	0.796	0.771	0.117	1.712
7. Knight et al., 2000	3	194.029	74	2.622	0.075 [0.062, 0.088]	0.930	0.914	0.084	1.063
8. Whitlatch et al., 1991	2	718.823	134	5.364	0.124 [0.115, 0.132]	0.760	0.726	0.114	1.777
9. Barreto-Osorio et al., 2015	4	649.076	203	3.197	0.088 [0.080, 0.095]	0.863	0.844	0.103	1.438
10. Rueda et al., 2017	1	176.144	65	2.710	0.077 [0.064, 0.091]	0.949	0.939	0.071	0.977
11. Knight et al., 2000	1	916.289	209	4.384	0.109 [0.102, 0.116]	0.782	0.759	0.117	1.759

*SSχ^2^, Chi-squared Scaling-Shifted; df, degrees of freedom; RMSEA, Root Mean Square Error of Approximation; CI, confidence interval; CFI, Comparative Fit Index; TLI, Tucker-Lewis Index; SRMR, Standardized Root Mean Square Residual; WRMR, Weighted Root Mean Square Residual.*

According to the results presented in Table 3, the model of Hébert et al. (2000) and Rueda et al. (2017) has the best goodness-of-fit indices (χ^2^/df < 5, CFI > 0.90, TLI > 0.90, RMSEA < 0.08, SRMR < 0.08, and WRMR < 1). The 12-item model of Hébert et al. (2000) presented two related factors. The Personal strain factor was composed of three items (items 9, 17, and 18) and the Role strain factor had nine items (items 2, 3, 6, 7, 10, 11, 12, 13, and 22), where the correlation between the factors was 0.998 and the factor loadings were between 0.350 (item 18) and 0.787 (item 12). On the other hand, the 13-item model of Rueda et al. (2017) presented a unidimensional structure (items 2, 3, 6, 9, 9, 10, 11, 12, 12, 13, 16, 17, 18, 19, and 22), with factor loadings ranging from 0.332 to 0.788 (Table 4).

**TABLE 4:** Factor loading and results of Andrich’s Rasch model.

Item	Factor loading	Outfit	Infit
2	0.522	1.010	1.080
3	0.722	0.661	0.703
6	0.639	0.834	1.040
9	0.699	0.855	0.964
10	0.636	0.805	0.988
11	0.665	0.852	0.875
12	0.778	0.720	0.940
13	0.788	0.732	0.839
16	0.332	1.180	1.160
17	0.725	0.793	0.865
18	0.398	1.180	1.200
19	0.509	1.080	1.030
22	0.660	0.822	0.882

### Reliability

Reliability was analyzed through the internal consistency method with the ordinal omega coefficient. The two-factor related model of Hébert et al. (2000) presented reliability problems in the Personal strain factor (ω = 0.546), unlike the Role strain factor (ω = 0.820). Thus, the Hébert et al.’s (2000) model was discarded for further analyses. On the other hand, the unidimensional model of Rueda et al. (2017) presented a good level of reliability of the scores (ω = 0.871; 95% CI: 0.842, 0.902). Complementarily, inter-item polychoric correlations were analyzed (Table 5), where the coefficients were found to be between 0.07 and 0.69 (*M* = 0.37, SD = 0.14).

**TABLE 5:** Matrix of inter-item polychoric correlations.

Item	2	3	6	9	10	11	12	13	16	17	18	19	22
2	–												
3	0.54	–											
6	0.25	0.44	–										
9	0.33	0.51	0.47	–									
10	0.38	0.50	0.40	0.45	–								
11	0.38	0.43	0.40	0.55	0.33	–							
12	0.31	0.50	0.49	0.53	0.48	0.56	–						
13	0.28	0.45	0.52	0.51	0.48	0.51	0.69	–					
16	0.11	0.23	0.26	0.21	0.11	0.20	0.17	0.17	–				
17	0.33	0.42	0.49	0.52	0.39	0.53	0.55	0.62	0.37	–			
18	0.18	0.23	0.32	0.19	0.34	0.24	0.26	0.32	0.42	0.23	–		
19	0.07	0.34	0.29	0.27	0.36	0.33	0.31	0.52	0.20	0.40	0.36	–	
22	0.37	0.55	0.42	0.50	0.47	0.36	0.56	0.40	0.17	0.43	0.13	0.35	–

### Evidence Based on Relations to Other Variables

Regarding convergent evidence, the model of the relationship between the ZBI and the CASE presented an adequate fit (χ^2^ = 368.820, df = 188, χ^2^/df = 1.962, CFI = 0.942, TLI = 0.935, RMSEA = 0.058 [90% CI: 0.049, 0.067], SRMR = 0.081, and WRMR = 1.068). The relationship between ZBI and CASE was significant and of strong degree (*r* = 0.667; *p* < 0.001; *r*^2^ = 0.445), sharing 45% of their variability. On the other hand, the AVE of the ZBI was 0.404, considered an adequate value for a unidimensional structure and a good level of reliability of the scores (Moral, 2019).

Regarding the discriminant evidence, the HTMT ratio of the ZBI with the other instruments was 0.607, considered an acceptable level, lower than 0.85. In addition, the square root of the AVE for the ZBI (0.634) was higher than the correlation between the ZBI and CASE (0.667). Therefore, considering the results obtained, it is possible to conclude that the ZBI scores have evidence of validity based on the relationship with other variables (convergent and discriminant evidence).

### Andrich Rasch Model (Rating Scale)

The unidimensional model of Rueda et al. (2017) presented a good overall fit to Andrich’s Rasch model (RMSEA = 0.078, TLI = 0.930, CFI = 0.899). Likewise, the fit values for the items, Outfit, and Infit were found to be between 0.70 and 1.30 (Table 4), which were considered satisfactory. On the other hand, Andrich’s Rasch model allowed the estimation of reliability under this perspective, presenting marginal reliability of 0.858 (Thissen and Wainer, 2001) and empirical reliability of 0.808, both considered good levels of reliability.

## Discussion and Conclusion

The caregiver burden is a common event among people caring for others with disabilities. Zarit et al. (1980) proposed the ZBI, as an instrument to evaluate this experience. To date, different versions of this measure have been developed for some conditions (i.e., dementia, Alzheimer, and cognitive complaints). The present study was conducted to assess the psychometric properties of the ZBI in Peruvian caregivers of people with intellectual disabilities. To our knowledge, this is the first study in this sample. To this end, we evaluated the factorial structure of ZBI, its internal consistency, and its association to other variables.

### Internal Structure

Regarding its internal structure, we assess the dimensionality of the previous nineteen models. Results from CFA indicated that Whitlatch et al.’s (1991) two-factor with 22 items model provided a poor fit to the data. These results are consistent with other studies (Knight et al., 2000; Li et al., 2018; Landfeldt et al., 2019). Thus, we opted to try other solutions. In the current sample, we found a significant improvement for Hébert et al.’s (2000) and Rueda et al.’s (2017) model, which have single- and two-factor structures, respectively. However, our data fit slightly better for Rueda et al.’s (2017) model.

Similar findings were obtained by Tartaglini et al. (2020), who found that the fit of the model improved by reducing to 17 items loaded on one factor, so they remained five more items (i.e., item 1: “Feel your relative asks for more help than he/she needs,” item 4: “Feel embarrassed over your relative’s behavior,” item 5: “Feel angry when you are around your relative,” item 8: “Feel your relative is dependent on you,” and item 14: “Feel that your relative seems to expect you to take care of him/her as if you were the only one he/she could depend on”). Four of these five items had the weakest factor loadings in Tartaglini et al. (2020), and four of five of these items were also eliminated for Rueda et al.’s (2017) model. Regarded item content, Rasch modeling confirmed that the data fit well for Rueda et al.’s (2017) model. None of the items reported severe misfit, which demonstrated unidimensionality. In addition, the Andrich Rasch model showed a better fit than the 12-item version of Pinyopornpanish et al. (2020), which did not demonstrate unidimensionality.

On the other hand, the results also showed a poor fit for the three and four-factor models. This finding contrasts with previous studies that revealed the ZBI is multidimensional. In this sense, Pinyopornpanish et al.’s (2020) validation study found that three-factor and four-factor provided a better fit to the data for ZBI-22 than a unidimensional model. It is noteworthy that the authors allowed the covariance of item 11 (Feel you do not have as much privacy as you) and item 12 (Feel your social life has suffered due to caring for your relative) residuals for all models (i.e., one-factor, two-factor, three-factor, and four-factor), which was needed to reach an acceptable fit. However, the specification of correlations between residuals may hide a misspecified model or a bad internal structure, showing an increase in goodness-of-fit indices, which would not contribute to the understanding of the model and the measurement of the construct (Dominguez-Lara, 2019).

Therefore, it can be concluded that, in informal caregivers of persons with intellectual disabilities, the use of a short version of the ZBI is better than the full 22-item version (ZBI-22). Specifically, in this study, the 13-item version proposed by Rueda et al. (2017) is suggested. The nine items that presented problems (1, 4, 5, 7, 8, 8, 14, 15, 20, and 21) do not contribute to the measurement of the construct “burden.” Item 1 (Feel that your relative asks for more help than he/she needs) because people with intellectual disabilities require a higher level of support for certain tasks, caregivers may not consider that these people exaggerate in their demands. Items 4 (Feel embarrassed over your relative’s behavior), 5 (Feel angry when you are around your relative), and 7 (Afraid what the future holds for your relative) were shown to be problematic as they link caregivers’ emotions to the present or future of people with intellectual disabilities, this possibly because of the familial bonding that occurs in most of them.

The situation pointed out in the previous items may be the same as with items 20 (Feel you should be doing more for your relative) and 21 (Feel you could do a better job in caring for your relative), related to feelings of guilt and inferiority on the part of the caregivers. Items 8 (Feel your relative is dependent on you), and 14 (Feel that your relative seems to expect you to take care of him/her as if you were the only one he/she could depend on) are related to the dependence of people with intellectual disabilities and caregivers, which is assumed in most cases, because of the family bond. Finally, item 15 (Feel that you do not have enough money to take care of your relative in addition to the rest of your expenses) is the one that relates little to the rest of the ZBI items, as it refers to a socio-economic aspect of the caregiver, so it seems to be a very specific issue within the instrument.

### Reliability

Regarding the reliability of the ZBI, we found that internal consistency for the personal train factor of Hébert et al.’s (2000) model was particularly low (<0.60), In contrast, the reliability for Rueda et al.’s (2017) model was adequate (>0.80). Our finding supports that, in this Peruvian sample, it is not necessary to split the ZBI into two distinct factors. These findings are very similar to the Argentinian study (Tartaglini et al., 2020) and to the Colombian sample (Rueda et al., 2017). The level of reliability obtained in the present study (ω = 0.87) was similar to that reported by Barros et al. (2019) with the 22-item version of the ZBI (α = 0.90). Likewise, the reliability estimation through the Andrich Rasch model also showed acceptable values (>0.80), similar to those obtained in the 12-item version of Pinyopornpanish et al. (2020). The results allow us to conclude that the ZBI scores present a good level of reliability from the Classical Test Theory and Rasch Measurement Theory.

### Relationship With Other Variables

According to theory, people who are caregivers tend to experience various emotional and social problems (Sherwood et al., 2005). In this sample, caregiver burden displayed a significant positive association with a measure of the risk of mistreatment, a result that is consistent with previous studies (Gimeno et al., 2021), which showed a strong correlation between ZBI and CASE (>0.80). In addition, the present study found a higher correlation between the CASE and ZBI total scores (*r* = 0.667) compared to that reported by Rivera-Navarro et al. (2018), who showed a moderate correlation (*r* = 0.486) in a sample of caregivers of people with dementia. On the other hand, the AVE of the ZBI was 0.404, adequate for the context of the study (Moral, 2019), and the discriminant evidence indicators showed acceptable levels. Thus, the ZBI scores present convergent and discriminant evidence.

### Strengths and Limitations

In interpreting the results from the study, several limitations are noteworthy. First, the sample was predominately female, so we were not able unable to examine the measurement invariance across gender. However, previous research has suggested that levels of experienced burden are different between women and men (Lai, 2012; Lin et al., 2017). Second, the burden was evaluated using a self-reported instrument. Thus, results could be affected by social desirability or memory biases (Althubaiti, 2016). Future research may also include other strategies as in-depth qualitative interviews. Third, we employed a convenience sample, so the results are not necessarily representative of the population. Fourth, the study design was cross-sectional, and it was not viable to assess test-retest reliability and predictive validity. Future studies using longitudinal designs could make available more useful information.

Despite the limitations, our study is the first to apply the ZBI to caregivers of people with intellectual disabilities. Moreover, the present work is the first to validate the Peruvian version of the ZBI. Furthermore, the unidimensional structure of the ZBI allows researchers and clinicians to use it and obtain an overall score easily, due to it consists of only 13 items. From a methodological perspective, this study assessed the internal structure using both Classical Test Theory and Rasch model and provided a model that explains the relationship of the ZBI with other psychological constructs using SEM. In this sense, our findings support the robust validity and reliability of the ZBI.

### Conclusion

The ZBI is a commonly used tool in international caregiver burden investigation. The key contribution of this study relies on the validation of the ZBI in a sample of caregivers of people with intellectual disabilities. Analysis of internal structure validity using CFA and Rasch modeling supports a unidimensional structure. Hence, this version of the ZBI allows for a time-efficient and useful assessment of the burden.

## Data Availability Statement

The raw data supporting the conclusions of this article will be made available by the authors, without undue reservation.

## Ethics Statement

The studies involving human participants were reviewed and approved by the Universidad César Vallejo. Written informed consent to participate in this study was provided by the participants’ legal guardian/next of kin.

## Author Contributions

AB-C and AT-M contributed to the conceptualization and design of the methodology of the study. AB-C provided study materials and acquired financial funding support for the project. AT-M organized the database and performed the data curation and formal analysis. AB-C and RP-A wrote the first draft of the manuscript. AB-C, RP-A, and AT-M wrote sections of the manuscript. All authors contributed to the manuscript revision, read, and approved the submitted version.

## Conflict of Interest

The authors declare that the research was conducted in the absence of any commercial or financial relationships that could be construed as a potential conflict of interest.

## Publisher’s Note

All claims expressed in this article are solely those of the authors and do not necessarily represent those of their affiliated organizations, or those of the publisher, the editors and the reviewers. Any product that may be evaluated in this article, or claim that may be made by its manufacturer, is not guaranteed or endorsed by the publisher.
